# Propagation of Spermatogonial Stem Cell-Like Cells From Infant Boys

**DOI:** 10.3389/fphys.2019.01155

**Published:** 2019-09-19

**Authors:** Lihua Dong, Stine Gry Kristensen, Simone Hildorf, Murat Gul, Erik Clasen-Linde, Jens Fedder, Eva R. Hoffmann, Dina Cortes, Jorgen Thorup, Claus Yding Andersen

**Affiliations:** ^1^Laboratory of Reproductive Biology, Copenhagen University Hospital, Copenhagen, Denmark; ^2^Department of Pediatric Surgery, Copenhagen University Hospital, Copenhagen, Denmark; ^3^Department of Urology, Aksaray University School of Medicine, Aksaray, Turkey; ^4^Department of Pathology, Copenhagen University Hospital, Copenhagen, Denmark; ^5^Centre of Andrology and Fertility Clinic, Department D, Odense University Hospital, Odense C, Denmark; ^6^Research Unit of Human Reproduction, Institute of Clinical Research, University of Southern Denmark, Odense, Denmark; ^7^Center for Chromosome Stability, Department of Molecular and Cellular Medicine, Faculty of Health and Medical Sciences, University of Copenhagen, Copenhagen, Denmark; ^8^Department of Pediatrics, Copenhagen University Hospital Hvidovre, Copenhagen, Denmark; ^9^Faculty of Health and Medical Sciences, University of Copenhagen, Copenhagen, Denmark

**Keywords:** cryptorchidism, fertility cryopreservation, *in vitro* expansion, male infertility, spermatogonial stem cell

## Abstract

**Background:**

Gonadotoxic treatment of malignant diseases as well as some non-malignant conditions such as cryptorchidism in young boys may result in infertility and failure to father children later in life. As a fertility preserving strategy, several centers collect testicular biopsies to cryopreserve spermatogonial stem cells (SSCs) world-wide. One of the most promising therapeutic strategies is to transplant SSCs back into the seminiferous tubules to initiate endogenous spermatogenesis. However, to obtain sufficient numbers of SSC to warrant transplantation, *in vitro* propagation of cells is needed together with proper validation of their stem cell identity.

**Materials and Methods:**

A minute amount of testicular biopsies (between 5 mg and 10 mg) were processed by mechanical and enzymatic digestion. SSCs were enriched by differential plating method in StemPro-34 medium supplemented with several growth factors. SSC-like cell clusters (SSCLCs) were passaged five times. SSCLCs were identified by immunohistochemical and immunofluorescence staining, using protein expression patterns in testis biopsies as reference. Quantitative polymerase chain reaction analysis of SSC markers LIN-28 homolog A (LIN28A), G antigen 1 (GAGE1), promyelocytic leukemia zinc finger protein (PLZF), integrin alpha 6 (ITGA6), ubiquitin carboxy-terminal hydrolase L1 (UCHL1) and integrin beta 1 (ITGB1) were also used to validate the SSC-like cell identity.

**Results:**

Proliferation of SSCLCs was achieved. The presence of SSCs in SSCLCs was confirmed by positive immunostaining of LIN28, UCHL1 and quantitative polymerase chain reaction for LIN28A, UCHL1, PLZF, ITGA6, and ITGB1, respectively.

**Conclusion:**

This study has demonstrated that SSCs from infant boys possess the capacity for *in vitro* proliferation and advance a fertility preservation strategy for pre-pubertal boys who may otherwise lose their fertility.

## Introduction

Cryptorchidism occurs with a frequency of around 3% in full-term pregnancies and in 30% of boys born prematurely. During the first year of life, the testes may spontaneously descend to the scrotum, but in total 1–3% of boys in the western world will undergo surgery to get their testes positioned in scrotum (Barthold and Gonzalez, 2003). Around one third of boys with cryptorchidism, especially bilateral cryptorchidism, suffer from infertility in adult life despite the presence of spermatogonial stem cells (SSCs) in the majority of testes biopsies (Hadziselimovic and Herzog, 2001). Another group of young boys in need of fertility preservation is those suffering from childhood cancer. Gonadotoxic treatments frequently destroy the entire population of SSCs and leave the boy infertile, however, the survival rates of cancer survival are above 80% with the evolution of toxic treatments (Chow et al., 2016). Therefore, prior to chemotherapeutic treatment this group of patients may benefit from having a biopsy excised and frozen from which SSCs can be isolated and, in theory, auto-transplanted after remission. Due to this potential, reproductive centers worldwide currently freeze testis biopsies to preserve SSCs and the germ cell niche.

Spermatogonial stem cells – a sub-population of undifferentiated spermatogonia – are required to sustain spermatogenesis by balancing self-renewal and differentiation in adults (Kanatsu-Shinohara and Shinohara, 2013). In primates, undifferentiated spermatogonia consist of type A dark (A_d_) and pale (A_p_) spermatogonia distinguished by hematoxylin staining. A_d_ spermatogonia contain a non-staining rarefaction-zone in nucleus, which is stained darkly. In contrast, A_p_ spermatogonia nuclei stain lightly and evenly. As there was no significant proliferation activity of A_d_ spermatogonia, Clermont and co-workers proposed that A_d_ and A_p_ spermatogonia are the reserve and the active stem cells, respectively (Clermont, 1969). Besides, the A_d_ spermatogonia are considered as true stem cells that generate A_p_ spermatogonia slowly (Ehmcke et al., 2006). A longitudinal study on cryptorchid boys indicates that the A_d_ spermatogonia are the key elements in establishing spermatogenesis (Hadziselimovic and Herzog, 2001). The authors compared the histological patterns of cryptorchid testes biopsied from boys younger than 2 years old boys with their counterpart sperm samples in adulthood. When A_d_ spermatogonia were present in the juvenile testis, 94% of the men had a normal sperm count. In contrast, in the absence of A_d_ spermatogonia, only 8% of patients had normal sperm count despite successful early surgery. Kraft et al. (2012) also reported a significant association between an abnormal A_d_ spermatogonia count at the time of orchiopexy and decreased sperm density in adulthood.

In 1994, the first report showing that murine SSCs transplantation restore spermatogenesis and generate functional sperm that give rise to normal offspring (Brinster and Avarbock, 1994; Brinster and Zimmermann, 1994). Subsequently, SSC transplantation has been successful in a variety of species such as pig, bovine and monkey (Honaramooz et al., 2002; Schlatt et al., 2002; Izadyar et al., 2003). In addition, transplanted SSCs from humans or other species can migrate onto the basal membrane of seminiferous tubules in recipient mice (Hermann et al., 2007; Izadyar et al., 2011; Zohni et al., 2012; Dovey et al., 2013). Critically, there is currently no data from transplanting SSCs to humans. Several obstacles need to be solved in order to approach clinical trials including the generation of a sufficient number of SSCs to warrant transplantation whilst avoiding transmission of potentially malignant cells from the cryopreserved tissue. Due to the small testis biopsies, it is necessary to develop propagation protocols while maintaining SSC identity.

In this report, we have derived and propagated SSC-like cells *in vitro* from a minute amount of testicular tissue obtained in infant boys, who underwent surgery for cryptorchidism. We observed the formation of spermatogonial stem cell-like cell clusters (SSCLCs), which could be passaged five times. We analyzed colonies by immunostaining and quantitative polymerase chain reaction (qPCR) using different SSC markers to demonstrate the potential expansion of A_d_ spermatogonia that retain expression of SSC markers, such as Lin-28 homolog A (LIN28), Ubiquitin carboxy-terminal hydrolase L1 (UCHL1) in the culture.

## Materials and Methods

### Human Testis Materials

Testis samples were obtained from five patients undergoing orchidopexy for unilateral and bilateral cryptorchidism. None of these boys had received chemotherapy, radiotherapy or hormonal treatment. Testicular biopsies around 5 mm^3^ were cryopreserved in 1.5 M ethylene glycol, 100 mM sucrose, 10 mg/ml human serum albumin (CSL Behring, Germany) in PBS buffer and stored at – 196°C (Kvist et al., 2006). Our center is certified by the Danish authorities to perform this treatment according to the European Union tissue directive. The frozen-thawed materials as well as fresh materials were used for SSC culture. For qPCR analysis, adult testis biopsies were obtained from healthy fathers who underwent vasectomy and one testis biopsy from a patient with Klinefelter syndrome. One additional unilateral undescended infant testis biopsy was examined using qPCR.

### Testicular Cell Isolation, Culture, and Cryopreservation

Fresh or thawed testicular biopsies weighing 5–10 mg were enzymatically digested using 450 U/mL collagenase type I (Worthington), 450 U/mL Hyaluronidase type II (Sigma), and 500 U/mL Trypsine TRL3 (Worthington) to prepare a cell suspension, as described previously (van Pelt et al., 1996). Testicular cells were collected and cultured overnight in uncoated dishes in supplemented alpha-modified MEM media, 15 mM 4-(2-hydroxyethyl)-1-piperazineethanesulfonic acid (HEPES), penicillin (100 IU/mL)-streptomycin (100 μg/mL) containing 10% FCS at 37°C and 5% CO_2_. After overnight incubation, floating cells were collected by centrifuging the media and the cell pellet was resuspended and cultured in uncoated dishes with supplemented StemPro-34 (composition see Supplementary Table 1). The cells were cultured in Nunclon Delta surface plates (Thermo Fisher Scientific) at 37°C in a humidified atmosphere with 5% CO_2__._ The SSCLCs were passaged with Accutase (Invitrogen) every 2–4 weeks to one new dish. Some SSCLCs were cryopreserved at – 196°C using vitrification methods. In principle, 2 – 5 cell clusters were collected and transferred through the vitrification medium-1 containing 10% DMSO (Sigma), 10% ethylene glycol (Merck), and 80% completed Stempro-34 culturing media to the vitrification medium-2 containing 20% DMSO, 20% ethylene glycol, 5 M sucrose and 60% completed Stempro-34 culturing media. The cell clusters were loaded into an open pulled straw (Minitube) and submerged into liquid nitrogen.

### Tissue Preparation and Staining and Germ Cell Number Counting

Testis tissue samples were fixed in Stieve’s solution for 24 h at room temperature, embedded in paraffin, and sectioned at 2-μm. The histological sections were stained with hematoxylin and eosin (HE), D2-40 (1:25, M3619, Dako) and placental-like-alkaline phosphatase (1:200, PL8-F6, Biogenex). The number of germ cells per tubular transverse section (G/T) and the number of A_d_ spermatogonia per tubular transverse section were measured based on in PAS stained sections as well as immunohistochemical staining of CD99, D2-40, C-KIT, OCT4, and PLAP (Dong et al., 2019). For each patient, at least 100 cross-sectional tubules were examined.

### Cell Cluster Preparation and Immunostaining

Freshly collected cell clusters from different passages were embedded by alginate followed by 4% agarose, then fixed in 4% paraformaldehyde for 4 h to overnight, embedded in paraffin, and cut into 5 μm sections. The sections were placed on slides and dried on heating plate setting at 37°C for 1 h. The sections were stored at room temperature till analysis. The sections were deparaffinized, subjected to antigen retrieval treatment using TEG buffer (10 mM Tris, 0.5 mM EGTA, pH 9), and blocked for 0.5 h at room temperature in 1% BSA in TBS buffer (50 mM Tris, 150 mM NaCl, pH 7.6) before the primary antibodies were applied. The following antibodies diluted in the blocking buffer (1% BSA in TBS buffer) were used: a polyclonal rabbit anti-LIN28 antibody (diluted 1:200; Ab46020, Abcam), a mouse monoclonal anti-UCHL1 (diluted 1:100, sc-271639, Santa Cruz, CA, United States), a mouse monoclonal anti-Vimentin (diluted 1:100, sc-6260, Santa Cruz, CA, United States), a polyclonal rabbit anti-SOX9 antibody (diluted 1:200, AB5535, Millipore) and a polyclonal rabbit anti-ACTA2 antibody (diluted 1:100, ab5694, Abcam). The slides were washed with TNT buffer (100 mM Tris, 150 mM NaCl, pH 7.6, 0.5% Tween 20) three times (10 min per time). For immunefluorescence staining, the sections were stained with a FITC-conjugated donkey anti-mouse IgG antibody (diluted 1:500 in blocking buffer; Jackson ImmunoResearch) or Alexa Fluor 594 donkey anti-rabbit IgG antibody (diluted 1:500 in blocking buffer; Jackson ImmunoResearch) for 1 h at room temperature. The DNA was visualized using DAPI staining before mounting slides with ProLong Gold Antifade Mountant (Life Technology). For immunohistochemical staining, sections were submerged in 1.5% H_2_O_2_ in TBS buffer to quench endogenous peroxidase before applying blocking buffer. Signals were visualized on sections by incubation with the secondary antibodies, either rabbit anti-mouse-HRP or donkey anti-rabbit-HRP (Dako, Glostrup, Denmark, 1:100 in blocking buffer) for 10 min at room temperature and visualized by peroxidase reaction with 3,3′-diaminobenzidine tetrahydrochloride (Dako) for 1–2 min. The slides were mounted with Pertex^®^ Histolab. Microscopic slides were evaluated on a Zeiss Axiophot microscope mounted with a Leica DFC420C digital microscope camera, and images processed in LAS software V4.9.

### Gene Expression Analysis

Total RNA from the testicular tissues and single cell cluster derived from different patients were extracted with the RNeasy Kit (Qiagen) or Absolutely RNA Nanoprep Kit (Agilent Technologies) according to the manufacturer’s instructions, respectively. cDNA synthesis was performed with High Capacity cDNA Reverse Transcription Kit (Applied Biosystems) according to the manufacturer’s instructions. The gene expression was detected on the LightCycler 480 Instrument II (Roche Diagnostics). The reaction mix consisted of 2 μL of template cDNA, 5 μL of TaqMan universal PCR master mix (Applied Biosystems), 0.5 μL of TaqMan primer assays (Supplementary Table 2) and 2.5 μL of H_2_O to a final volume of 10 μL. A 96-well plate was used, and each sample was run in duplicate. The PCR cycling conditions were one initiation cycle at 95°C for 10 min, followed by 45 cycles of 95°C for 10 s, 60°C for 15 s, 70°C for 10 s, and finally, one cycle at 95°C for 1 min. The raw Ct value was reported from the Roche RCR software. The relative gene changes were calculated referring to healthy adult testis using GAPDH as an internal control. *P*-values were calculated by non-parametric One-way ANOVA using GraphPad Prism 7.0.

### Whole Mount Immunofluorescence Assay

Each single cluster was dissociated by incubation in 100 μl ready-to-use accutase for 10 min at 37°C, then seeded on 2% human serum albumin coated microscope glass cover slips (Propper) in a 24 well plate. New clusters formed after around 3 weeks. Clusters were washed three times with PBS and fixed with 100% cold methanol (–20°C pre-chilled) for 30 min, and then washed three times in PBS and stored at 4°C for several weeks or analyzed immediately. The clusters were blocked in 0.1% Triton-X100, 1% BSA TBS for 2 h at room temperature before the primary antibodies were applied. A polyclonal goat anti-human VASA antibody (diluted 1:100; AF2030, Novus Biologicals) was used. The following day each cluster was washed six times with TBS and incubated in TBS with an Alexa Fluor 594 donkey anti-goat IgG antibody (diluted 1:500; Jackson Immunoresearch) at 4°C overnight. The DNA was visualized using DAPI staining before mounting slides with ProLong Gold Antifade Mountant (Life Technology). Samples were analyzed with a Leica Microsystems. A Leica digital camera was used for analysis and image capture.

## Results

### Characterization of Germ Cells in the Undescended Testis From Infant Boys

**FIGURE 1:**
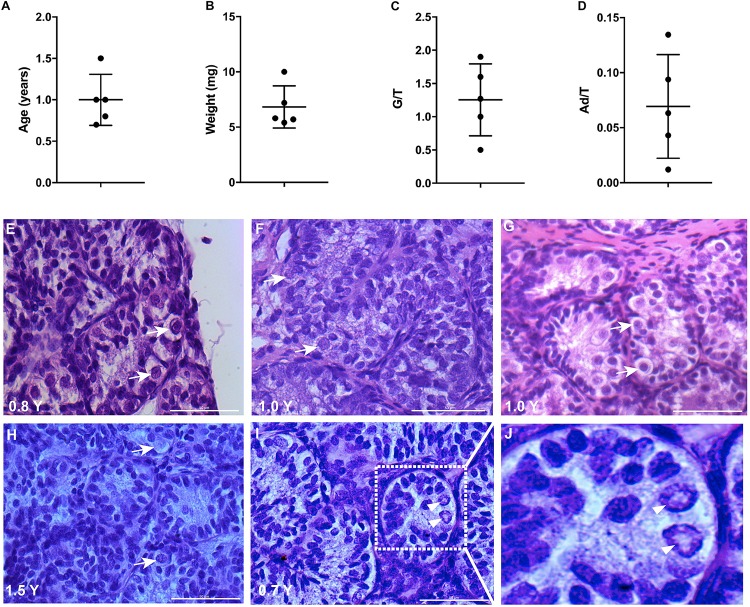
Testicular biopsies analysis for culture. **(A,B)** The age and weight of biopsies, error bars represent standard deviations. **(C,D)** Germ cell (G/T) number and A_d_ spermatogonia (Ad/T) number per tubular transverse section in five cryptorchid boys. Mean and standard deviations are shown. **(E–I)** Histological profile of testicular biopsies from five individual samples. Scale bar: 50 μm. White arrows indicate germ cells. **(J)**, higher magnification from **I** demonstrates the rarefaction zone in nuclear indicating A_d_ spermatogonia. Arrow heads indicate A_d_ spermatogonia.

The mean age of boys having a biopsy was 1 year (range 0.7–1.5 years old) and the weight of the biopsy was on average 7 mg (range 5–10 mg) (Figures 1A,B). All the testis biopsies contained germ cells and A_d_ spermatogonia with an average number of 1.3 and 0.07 per tubular transverse section, respectively (Figures 1C,D). The histological profiles are represented in Figures 1E–I and the A_d_ spermatogonia are presented in Figure 1J.

### SSCs Express LIN28 and UCHL1 in the Infant Testis

**FIGURE 2:**
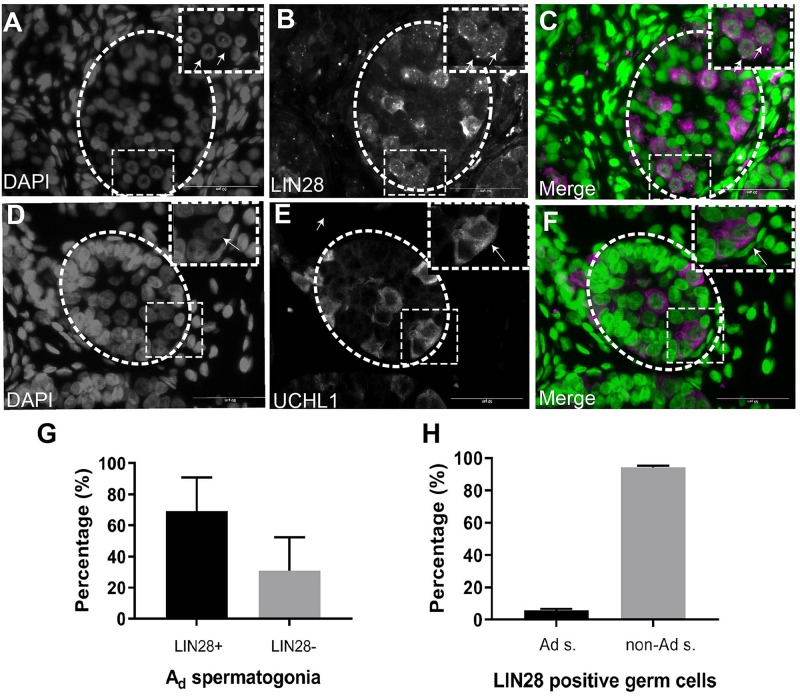
Immunofluorescence staining analysis of germ cells in the undescended testis. **(A)**, DAPI. **(B)**, LIN28. **(C)**, Merge (DAPI in green and LIN28+ in magenta). LIN28 positive signals are present in germ cells with rarefaction zone in the nuclei. Dash lines represent seminiferous tubules. Arrows showing the rarefaction zone in the germ cell nuclei. **(D)**, DAPI. **(E)**, UCHL1, **(F)**, Merge (DAPI in green and UCHL1+ in magenta). Dash lines represent seminiferous tubules. Arrows showing the rarefaction zone in the germ cell nuclei. Scale bar: 50 μm; **(G)**, Quantification of the percentage of LIN28 positive A_d_ spermatogonia (A_d_ s.) in total A_d_ spermatogonia. **(H)**, The percentage of A_d_ spermatogonia in total LIN28 positive germ cells in the undescended testis.

To investigate the molecular nature of the SSCs, we stained the infant testis with LIN28 and UCHL1 (PGP 9.5). LIN28 and UCHL1 were expressed in gonocytes and spermatogonia, including spermatogonia with rarefaction-zone in their nuclei (Figures 2A–F). Further quantification of LIN28 positive cells and A_d_ spermatogonia demonstrated that *LIN28* was expressed around 60% of A_d_ spermatogonia and 5% of LIN28 positive germ cells were A_d_ spermatogonia (Figures 2G,H).

### Derivation and Propagation of Human SSC-Like Cells From Infant Boys

**FIGURE 3:**
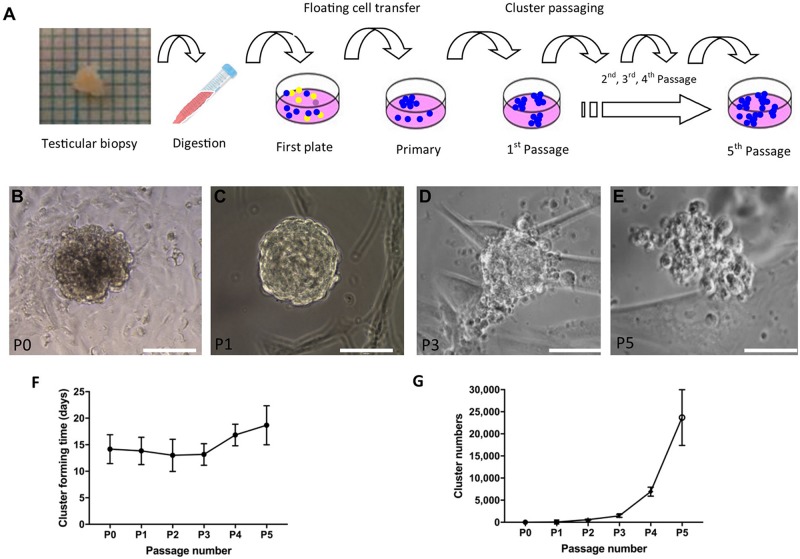
Derivation and propagation of human spermatogonial stem cell like cell clusters (SSCLCs) from infant boys. **(A)**, Methodology of deriving SSCLCs from testicular biopsies. One square is 1 mm^2^. **(B)**, SSCLCs forming from primary cell culture. **(C)**, First passage. **(D)**, Third passage. **(E)**, Fifth passage. Scale bar 100 μm. **(F)**, Cluster forming time after passaging (mean and standard deviations). **(G)**, Estimated SSCLC numbers (mean and standard deviations).

To propagate SSC *in vitro*, SSCs were the first enriched using a differential plating method from digested single-cell suspension (Figure 3A). After 2–3 weeks, cell clusters formed (Figure 3B). Four to 16 clusters formed in primary cell culture and clusters with a grape-like morphology were passaged. The maximum size of cell clusters was around 100–200 μm in diameter. During passaging, the clusters became less compact and large round cells became visible (Figures 3C–E). Low magnification picture of cell culture from different passages were shown in Supplementary Figure 1. The new clusters formed after around 2 weeks in new dishes, and the clusters were passaged five times in our setup (Figure 3F). Every passage was derived from 4 to 6 clusters after the first passage. The number of clusters was estimated to be more than five thousand from 4 passages (Figure 3G).

### Human SSC-Like Cells Express LIN28 and UCHL1

**FIGURE 4:**
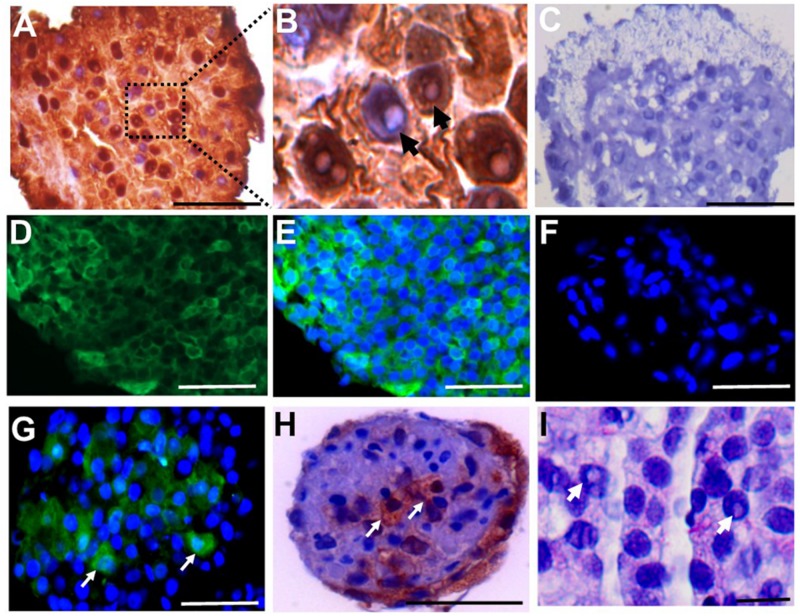
SSCLC containing LIN28 + and UCHL1 + cells with rarefaction zones. Immunostaining of cross sections of primary SSCLCs by **(A)**, anti-LIN28 in brown and hematoxylin staining shown in blue. Scale bar: 50 μm. **(B)**, higher magnification (100 × in microscope) of A illustrating rarefaction zones in nucleus. Arrows showing the rarefaction zones in the nuclei. **(C)**, negative control for IHC. Scale bar: 50 μm. **(D)**, UCHL1, Scale bar: 50 μm. **(E)**, UCHL1, and DAPI merged. Scale bar: 50 μm. **(F)**, Negative control for immunofluorescence staining of UCHL1. Scale bar: 50 μm. **(G)**, UCHL1 and DAPI merged cluster of passage 3. Green is UCHL1, blue is DAPI. Arrows showing the UCHL1 positive cells in SSCLCs. Scale bar: 50 μm. **(H)**, immunochemical staining of SSCLC against UCHL1. Arrows showing UCHL1 positive cells in the SSCLC of passage 3. Scale bar: 50 μm. **(I)**, PAS staining of SSCLC showing cells with rarefaction zones in passage 3. Scale bar: 10 μm. Arrows showing the rarefaction zones in the nuclei.

To investigate whether the cell clusters contained SSCs, colonies were immuno-stained with LIN28 and UCHL1. LIN28 was expressed in the primary clusters (Figures 4A–C). In addition, cells containing a rarefaction-zone in the nuclei were also visible by hematoxylin counterstaining, indicating the possible presence of A_d_ spermatogonia in the clusters (Figure 4B). UCHL1 was also expressed in SSCLCs (Figures 4D–F). The proportion of UCHL1 positive cells in SSC clusters was heterogeneous (Figures 4G,H). Rarefaction-zone in SSCLC was also shown by PAS staining (Figure 4I). Immunohistochemistry analysis of UCHL1 in adult testis as well as SSCLCs were shown in Supplementary Figure 2. These data suggest that SSCLCs contain cells which resemble the molecular and morphological characteristics of endogenous SSCs.

### qPCR Analysis of SSCLCs

**FIGURE 5:**
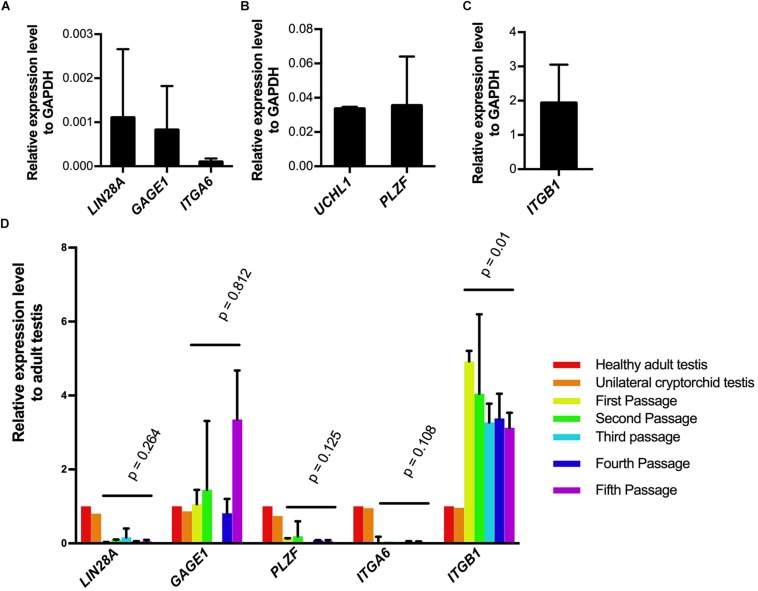
Germ cell marker expression analysis of SSCLCs. **A-C**, qPCR analysis showing the expression of germ cell markers LIN28A, UCHL1, GAGE1, ITGA6, ITGB1 relative expression level to GAPDH in primary SSCLCs. The bars show standard deviations. **(D)**, The relative expression levels of LIN28, GAGE, PLZF, ITGA6 and ITGB1 to adult testis and error bars in the SSCLCs of passage 1–5. *P*-values are showing the statistics analysis using one-way ANOVA.

To investigate whether SSCLCs express other adult SSC markers, we collected the SSCLCs for qPCR analysis. Along with analysis of *LIN28A* and *UCHL1*, we evaluated the other SSC expression gene markers, *PLZF* (*ZBTB16*), G antigen 1 (*GAGE1*), integrin subunit alpha 6 (*ITGA6*) and integrin subunit beta 1 (*ITGB1*) (Sadri-Ardekani et al., 2009). The level of mRNA of *LIN28A, UCHL1, GAGE1, PLZF*, *ITGA6*, and *ITGB1* relative to the house keeping gene glyceraldehyde-3-phosphate dehydrogenase (*GAPDH)* in primary SSCLCs was presented in Figures 5A–C. *UCHL1*, *PLZF* and *ITGB1* were highly expressed in SSCLCs compared to *ITGA6*, *LIN28A*, and *GAGE1*. To trace the gene expression dynamics during passaging relative to adult endogenous testicular expression level, the SSCLCs were collected and analyzed from five passages. *LIN28A, UCHL1, GAGE1, PLZF*, and *ITGA6* expression level did not change significantly during passaging. The expression level of *ITGB1* showed a statistically significant reduction (Figure 5D). Testis tissue from a Klinefelter patient without germ cells showed lower *LIN28A, GAGE, PLZF, ITGA6*, and *ITGB1* gene expression compared with normal adult testis tissue, indicating these markers are enriched in germ cells (Supplementary Figure 3).

### SSCLCs Maintain SSC-Like Identity After Vitrification and Long-Term Culture

**FIGURE 6:**
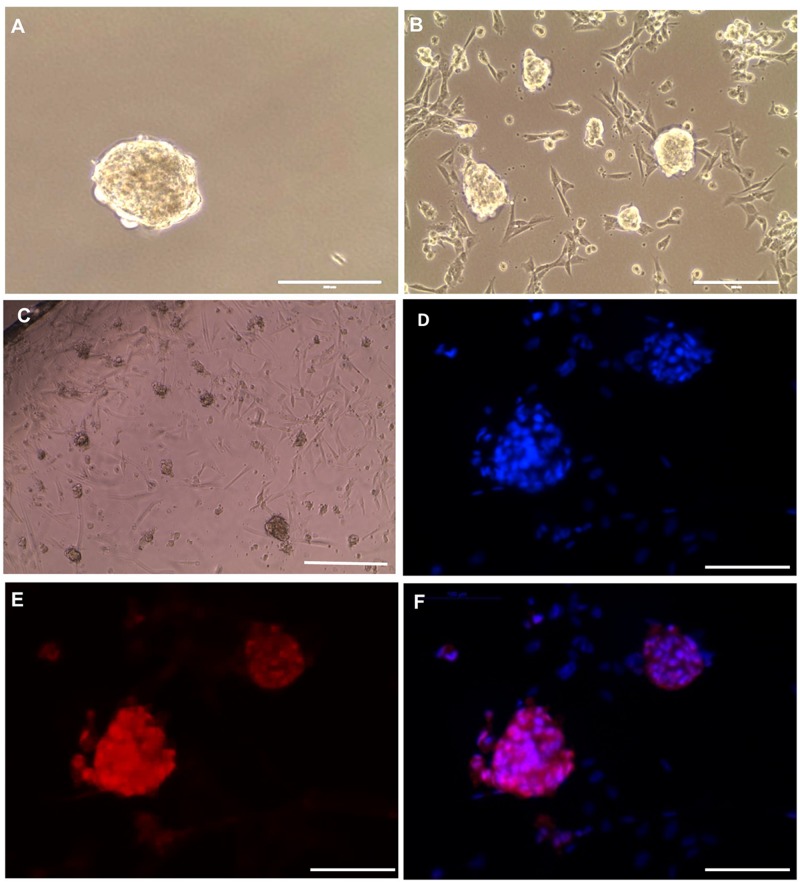
SSCLCs maintain their SSC-like identity after vitrification and long-term propagation **(A)**, Frozen cluster. **(B)**, Culture for 35 days of growth post warming. **(C)** Bright-field image of new clusters (passage 5), scale bar: 400 μm. **(D–F)** whole mount immunostaining of SSCLCs at passage 5, **D**, DAPI; **E**, VASA; **F**, Merge. Scale bar: 100 μm.

To preserve the *in vitro* propagated SSCLCs for clinical applications, vitrification method was used to cryopreserve SSCLCs. Warmed SSCLCs resumed proliferation and new SSCLCs formed after 35 days in culture (Figures 6A,B). To investigate the germ cell identity in the long-term culture, SSCLCs were passaged on coated glass cover slides, and new clusters formed after around 3 weeks in passage 5 (Figure 6C). The germ cell marker, VASA, positive immunofluorescence was detected in the clusters (Figures 6D–F). Negative control is shown in the Supplementary Figure 4. The VASA specificity was validated using adult health testis by co-staining of Sertoli cell marker SOX9 (Supplementary Figure 5). These data show that SSCLCs can be vitrified and warmed without significantly affecting their capacity for *in vitro* proliferation.

### SSCLCs Contains Somatic Cell Like Cells

**FIGURE 7:**
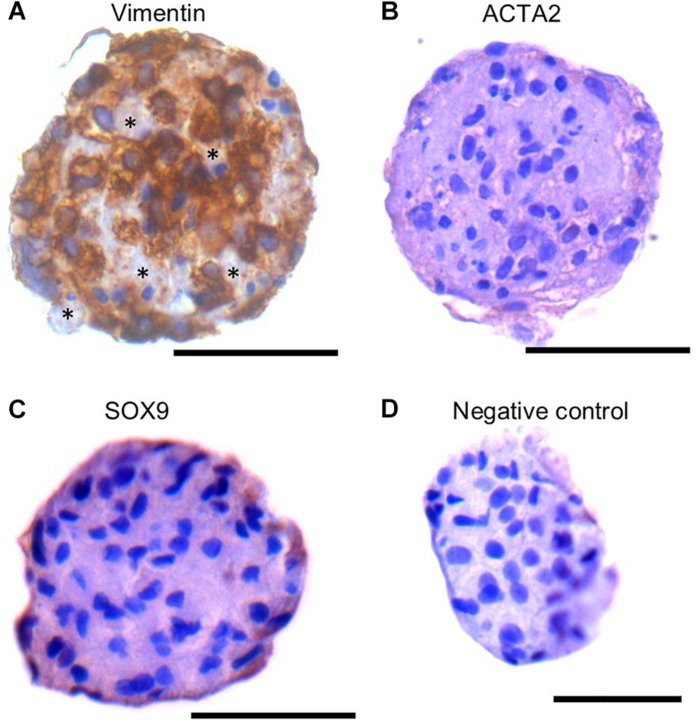
Mesenchymal cell markers in SSCLCs Immunohistochemical analysis of Vimentin **(A)**, ACTA2 **(B)**, SOX9 **(C)** and negative control **(D)**. Scale bar 50 μm. Stars represent Vimentin negative area or cells in SSCLC.

The SSC niche consists of other cell types, including mesenchymal cells. Since the SSCLCs were clearly not homogenous for SSC-like cells, we addressed whether our clusters contained somatic-cells such as mesenchymal cells using immunostaining against Vimentin, SOX9, and ACTA2 (Zheng et al., 2014). Vimentin was expressed in sub-population of SSCLCs, however, SOX9 and ACTA2 expressions were not detected in SSCLCs (Figures 7A–C). The positive controls for Vimentin, and ACTA2 staining are shown in Supplementary Figure 6. We also performed qPCR to detect the transcripts encoding Anti-Mullerian Hormone (AMH), which is expressed in Sertoli cells, Insulin-like 3 (INSL3) as a Leydig cell marker and fibroblast-specific protein 1 (FSP1) as a fibroblast cell marker. Whilst we detected amplified signal of FSP1 (Supplementary Figure 7), AMH and INSL3 were undetectable (data not shown). These findings suggest that in addition to SSCs the SSCLCs may contain mesenchymal cells as well as fibroblasts, consistent with the natural stem cell niche.

## Discussion

The present study demonstrates for the first time the establishment of human SSCLCs that can be propagated from small testis biopsies obtained in infant boys undergoing surgery for cryptorchidism. This is an important step forward in the development of a future fertility preserving strategy for very young boys at risk of losing capacity for fertility in adulthood. We managed to propagate numerous SSCLCs from as little as 5 mg testicular tissue. This quantity is 20 times lower than that used in previous studies, which used adult testis tissue (Sadri-Ardekani et al., 2009; Gat et al., 2017). Since transplantation of a suspension of cells from neonatal testis resulted in a high efficiency in mice (Shinohara et al., 2001), the propagation of SSCLCs from such small amounts of neonate testis tissue may be due to an enrichment of SSCs or a higher proliferation capacity of SSCs and its SSC precursors, gonocytes and pre-spermatogonia in infant boys. Our data indicate that the proliferative potential of SSCs from young boys may be greater than those obtained from adults and possess a hitherto undiscovered potential for proliferation *in vitro*.

The SSCLC identity was confirmed by expression of several SSC specific markers. We found SSCLCs contained cells, which displayed a nucleus with a rarefaction-zone, one of the unique features of A_d_ spermatogonia and considered to be the true SSC (Boitani et al., 2016). We also observed that some cells have two rarefaction-zones, which we did not observe in our testicular samples. However, two or three rarefaction zones in human A_d_ spermatogonia was also reported (von Kopylow et al., 2012). To our knowledge this is the first study to show that *in vitro* propagated cells contain a rarefaction-zone in the nucleus, the majority of which (60%) expressed LIN28. LIN28 has recently been demonstrated to be expressed in undifferentiated spermatogonia in several mammals including human (Zheng et al., 2009; Aeckerle et al., 2012; Lee et al., 2016; Ma et al., 2016). The SSCLCs also contain cells that express UCHL1, which is also expressed in spermatogonia of primates (Devi et al., 2015; von Kopylow and Spiess, 2017). The detection of mRNA encoding other SSC markers such as PLZF, ITGA6 and ITGB1 further confirmed the stem-like properties of the SSCLCs (Sadri-Ardekani et al., 2009; Sadri-Ardekani et al., 2011; von Kopylow and Spiess, 2017). These markers were consistently expressed in each passage, indicating the maintenance of the SSC-like cell identity in a long-term *in vitro* culture. The expression level of LIN28A is very low based on the qPCR analysis, although immunocytochemistry of LIN28 indicating the high expression level in SSCLCs. The reason is probably due to our antibody can recognize LIN28A and LIN28B, however, the qPCR results only indicated the expression level of LIN28A. Wu et al. (2009) identified that LIN28B is the main isoform of LIN28 in human SSC.

Although UCHL1, PLZF, and ITGA6 were considered specific to SSCs in testis, two recent papers have demonstrated that other testicular cells also show expression of those markers (Eildermann et al., 2012; Kossack et al., 2013). We have been able to confirm these results by showing qPCR expression in testis tissue from a Klinefelter patient. However, the expression level of the aforementioned genes was lower in the adult Klinefelter patient than normal adult testis tissue, suggesting those markers were expressed in SSCs.

Recently, human spermatogonia were intensively investigated by single cell RNA sequencing and revealed the high heterogeneity in neonatal as well as adult human testis (Guo et al., 2017, 2018; Wang et al., 2018; Sohni et al., 2019). It was previously understood that undifferentiated spermatogonia consisted of two cell types in the adult testis, A_d_ and A_*p*_ spermatogonia. Single cell RNA sequencing and bioinformatic analyses have revealed four type of undifferentiated spermatogonia in adult testis and three germ cell subsets in neonatal testis. UCHL1, PLZF and LIN28 are expressed both undifferentiated and differentiated spermatogonia (Sohni et al., 2019; Tan and Wilkinson, 2019). Because these transcripts are not specific for any subpopulation of undifferentiated spermatogonia, it is not possible to determine which subtype of spermatogonia is proliferating *in vitro*. The single cell sequencing needs to be performed from cultured cells to align the database of neonatal and adult testis and to characterize the proliferated SSCLC and its origins.

There have been several studies focused on propagation of human SSCs from adult testes. Sadri-Ardekani et al. (2009) were the first to report successful propagation of human SSC and showed germline stem cell-like cell colonies formation. Several groups subsequently have described the derivation of embryonic stem cell-like cells (Golestaneh et al., 2009; Kossack et al., 2009; Mizrak et al., 2010), clusters of human testicular fibroblast cells (Ko et al., 2010), or clusters of mesenchymal progenitors from primary cultures of human adult testis (Chikhovskaya et al., 2014). The morphology of the SSC-like clusters obtained in our study were grape-like cell aggregates, which contrast to those previously published articles where colonies showed more sharp-edged and compact embryonic stem cell-like colonies (Mizrak et al., 2010). Our approach to enrich SSCs by collecting floating cells may have allowed successful propagation of SSCLC, while other two other studies used the cells attached to the bottom of the culture dish for propagation, which formed testis like organoids or testis-cord like structures (Mincheva et al., 2018; von Kopylow et al., 2018). Indeed, floating cells have been also cultured in other two studies, however, the SSCs did not show long term survival in their culture systems and the predominant cells in the dishes were mesenchymal (stem) cells (Chikhovskaya et al., 2014; Zheng et al., 2014). Chikhovskaya’s culture media did not include human glial cell line-derived neurotrophic factor (GDNF) which is believed a central regulator for determination of undifferentiated spermatogonia. It was shown that gene-targeted mice with one GDNF-null allele showed depletion of stem cell reserves, whereas mice overexpressing GDNF showed accumulation of undifferentiated spermatogonia (Meng et al., 2000). The main differences of our culture system with Zheng’s system was that we subpassaged SSCLCs instead of passaging all cells from dish, based on the outcomes suggested by a primate study which showed the separation of somatic and germ cells is required to establish primate spermatogonial culture (Langenstroth et al., 2014).

In the present study, we estimated there was a 1000-fold increase in SSCLC number after three passages. It is currently unknown whether this will provide sufficient numbers of SSCs to warrant transplantation. However, we did not observe apparent signs of a reduced proliferative capacity of SSCLCs during passaging suggesting that further passaging is possible. This may be particularly important, since cultures of human SSC beyond three passages may diminish the potential contamination from cancer cells, due to the elimination of acute lymphoblastic leukemia cells from human testicular cell cultures after 14 days culture (Sadri-Ardekani et al., 2014).

There are several limitations of our study and some questions remain unanswered. The SSCs could not be obtained purely as there are some somatic-like cells including fibroblasts. Since we did not quantify the population of SSCs in SSCLCs during passaging and the fact that SSCLCs contain mesenchymal cells, the proliferation rate of SSCs could not be calculated precisely. In addition, we did not compare the freshly collected or thawed biopsy samples in terms of their profile of molecular characteristics. Furthermore, the functionality of the propagated SSCs was not tested. The xenotransplantation of dissociated cells from SSCLCs into nude mice should be performed to demonstrate the new colony formation in the mouse SSC niche.

In conclusion, we succeeded in long-term culture of human SSCLCs starting with small pieces of testicular tissue from infant boys. The presence of specific markers of early germ cells confirmed the presence of SSC-like cells which were sustained in culture for five passages. The results are encouraging for a continued research effort to develop a clinically acceptable solution for maintaining fertility in young boys in need of preserving fertility.

## Data Availability

The raw data supporting the conclusions of this manuscript will be made available by the authors, without undue reservation, to any qualified researcher.

## Ethics Statement

This study was carried out in accordance with the recommendations of Helsinki II declaration. The protocol was approved by the ethical committee of the Capital Region of Copenhagen (H-2-2012-060). All adult participants or the parents of participants under the age of 18 years received written information and provided their written informed consent.

## Author Contributions

LD conceived and designed the study, collected and assembled the data, analyzed and interpreted the data, and wrote and approved the final manuscript. SK conceived and designed the study, collected and assembled the data, analyzed and interpreted the data, and approved the final manuscript. SH collected and assembled the data, and approved the final manuscript. EC-L and JF provisioned the study material, analyzed and interpreted the data, and approved the final manuscript. MG collected the data, and approved the final manuscript. EH and DC analyzed and interpreted the data, and approved the final manuscript. CA conceived and designed the study, analyzed and interpreted the data, and wrote and approved the final manuscript. JT conceived and designed the study, provisioned the study material, analyzed and interpreted the data, and approved the final manuscript.

## Conflict of Interest Statement

The authors declare that the research was conducted in the absence of any commercial or financial relationships that could be construed as a potential conflict of interest.
