# Supplementary material for: Propagation of Spermatogonial Stem Cell-Like Cells From Infant Boys
Source: Front Physiol. 2019 Sep 19;10:1155. doi: 10.3389/fphys.2019.01155 (PMC6761273; doi:10.3389/fphys.2019.01155)
Supplement: Supplementary file 1 [file Data_Sheet_1.docx]

**Supplemental data**


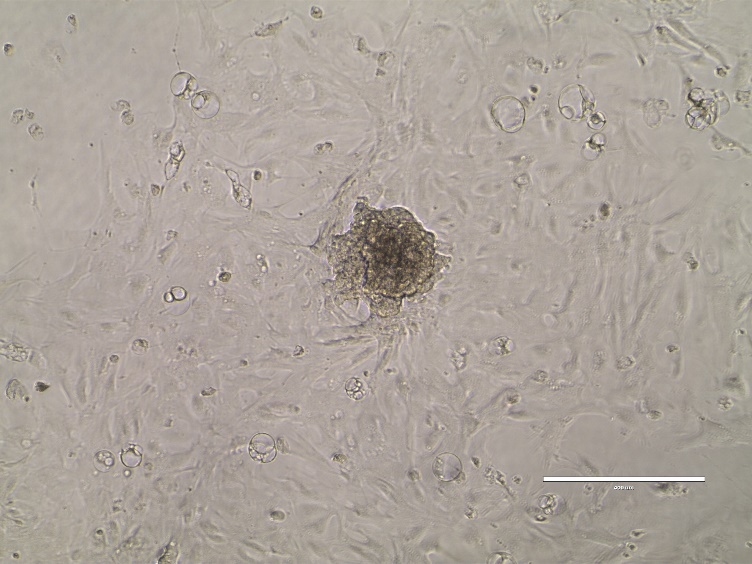
**Propagation of spermatogonial stem cells from infant boys**

**A**


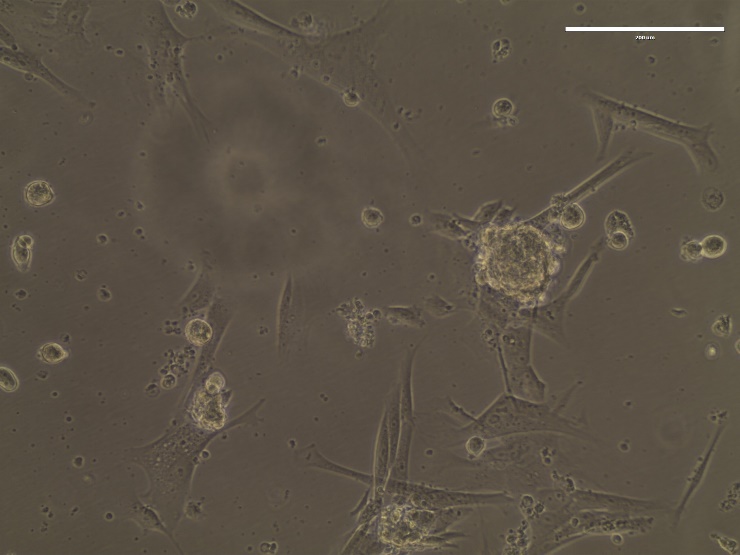


**B**


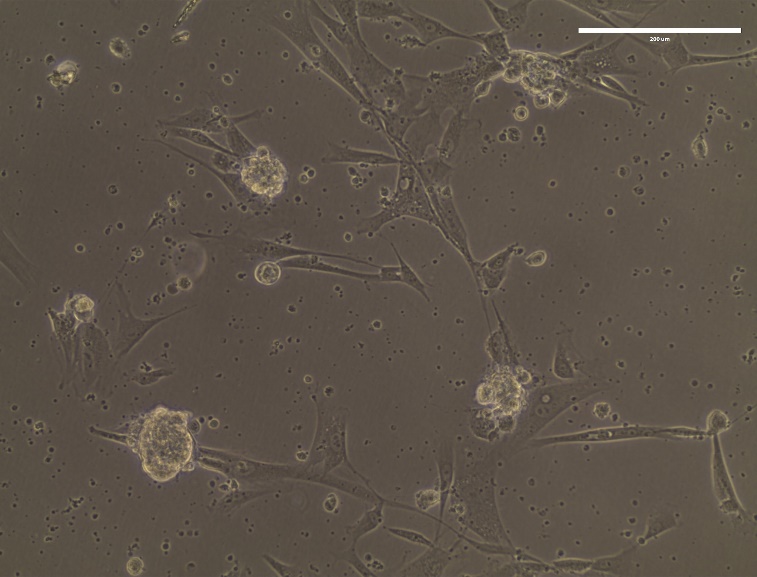

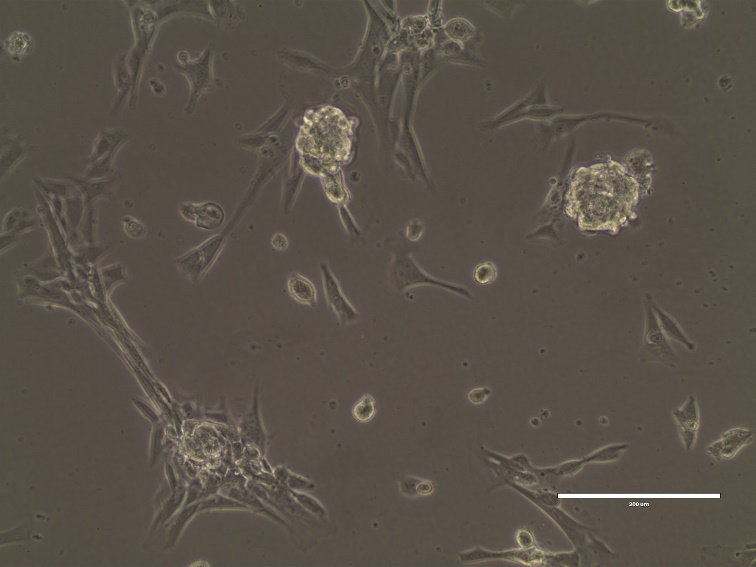


**D**

**C**

Supplemental figure 1 Low magnification of SSCLCs from different passages. A: Primary, B: first passage, C: third passage, D: fifth passage. Scale bars: 200 µm.


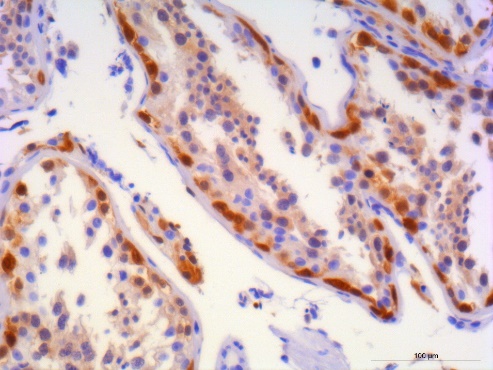

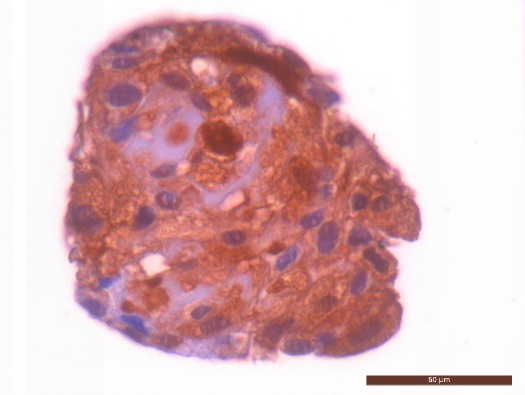


**B**

**A**

Supplemental figure 2 immunohistochemistry of UCHL1 on adult human testis (A), different SSCLCs (B). Scale bars: A, 100 µm; B, 50 µm.


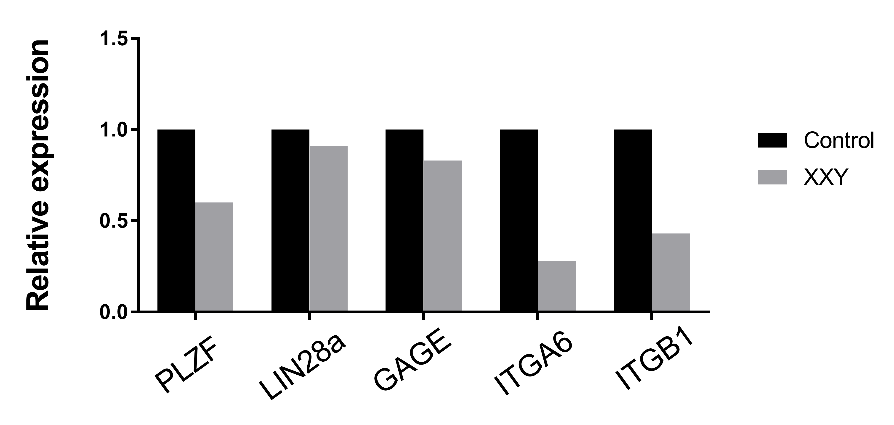


Supplemental Figure 3 qPCR analysis of the expression of PLZF, LIN28a, GAGE, ITGA6, ITGB1 in testis in healthy adults and in an adult man with Klinefelter syndrome (XXY syndrome). The value of the Klinefelter patient is the relative expression levels to adult healthy testes which serves as control testis.


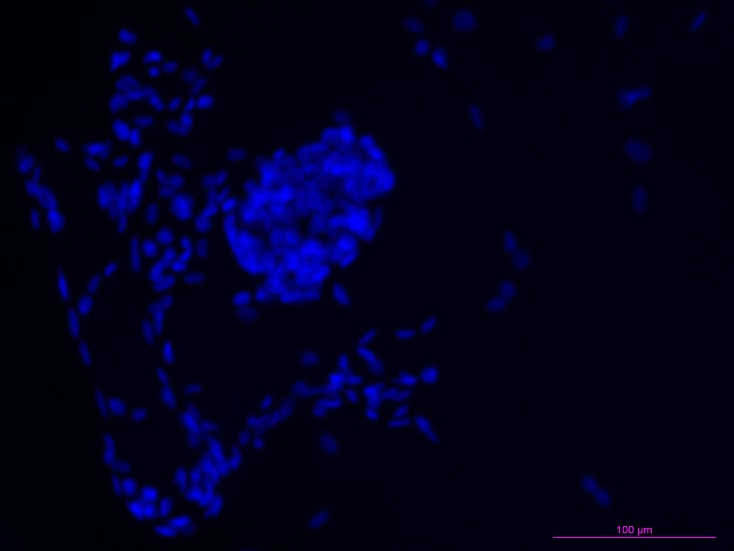


Supplemental Figure 4 Whole mount immunostaining negative control (DAPI and red channel merged), scale bar is 100 µm.


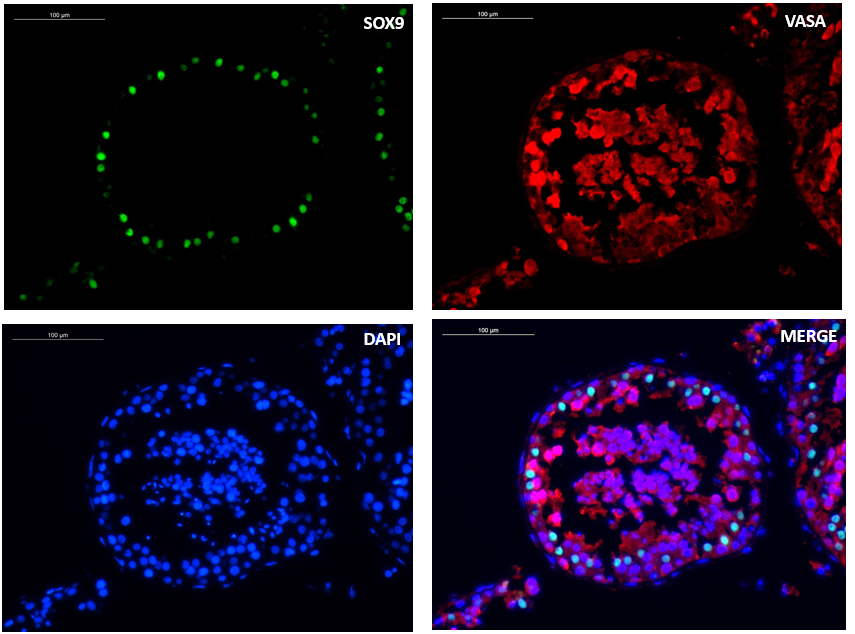


**D**

**C**

**B**

**A**

Supplemental Figure 5 Dual immunostaining of VASA and SOX9 in an adult healthy testis with the age of 36 years. A, SOX9; B, VASA; C, DAPI and D, Merge. scale bar is 100 µm.


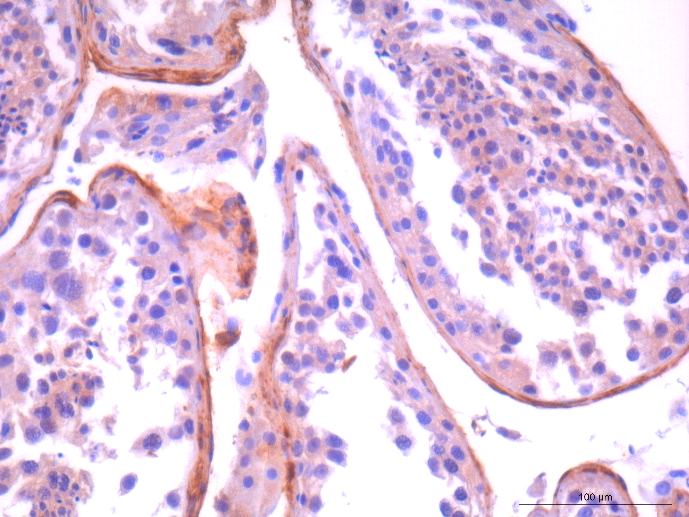

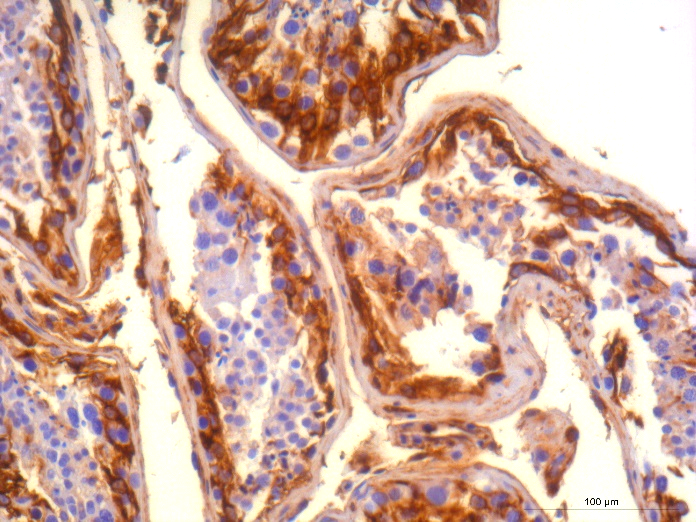


**ACTA2**

**Vimentin**

**B**

**A**

Supplemental Figure 6 Immunochemical staining of Vimentin (A) and ACTA2 (B) in an adult healthy testis with the age of 36 years. scale bar is 100 µm.


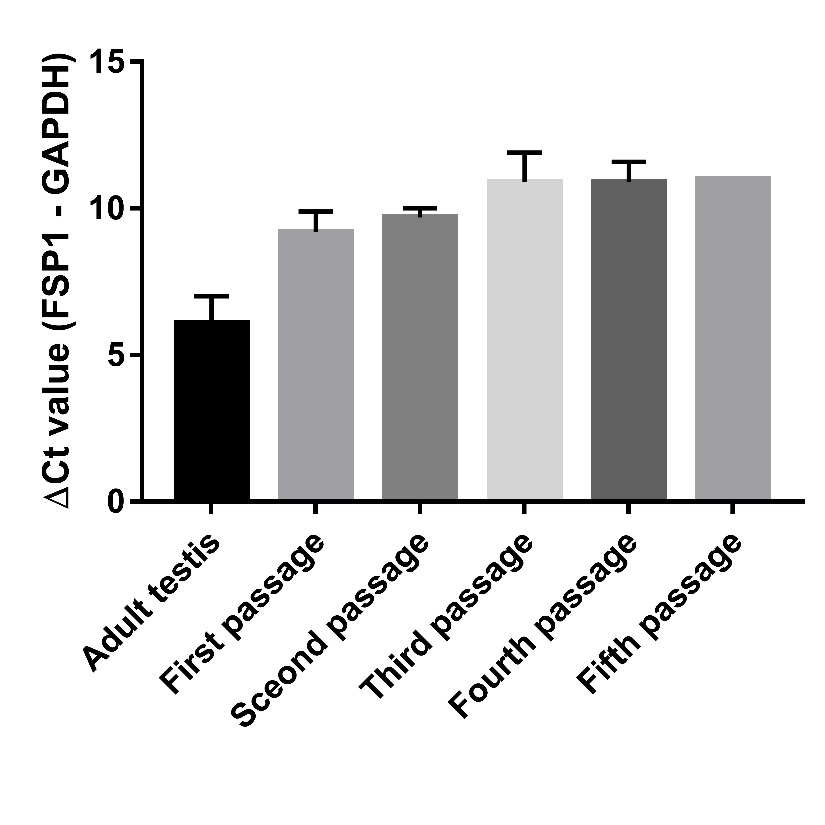


Supplemental Figure 7. The expression level of FSP1 in adult testis and passaging SSC clusters relative to housekeeping gene GAPDH. The bars express the standard derivations.

Supplemental table 1 The composition of supplemented StemPro media (Filtrated)

|  | **Company** | **Product Nr.** | **100 ml** |
| --- | --- | --- | --- |
| Stem Pro-34 SFM | Invitrogen | 10639-011 | 97.4 ml |
| Bovine Albumine | Roche | 10735094001 | 0.5 g |
| D(+) Glucose | Sigma | G7021 | 0.6 g |
| Ascorbic acid | Sigma | A4544 | 1.8 mg |
| d-biotin | Sigma | B4501 | 1 mg |
| Pyruvic acid (sodiumpyruvate) | Sigma | P2256 | 1 ml |
| 2-beta Mercaptoethanol | Sigma | M7522 | 100 ul |
| DL-Lactic acid | Sigma | L4263 | 100 ul |
| MEM-non essential AA | Invitrogen | 11140-035 | 1 ml |
| Insulin-transferrin-selen | Gibco | 41400-045 | 1 ml |
| Glutamax | Invitrogen | 25030-024 | 1 ml |
| Putrescine, 100 mg/ml | Sigma | P7505 | 10 ul |
| MEM Vitamine solution | Invitrogen | 11120-037 | 1 ml |
| b-Estradiol 0.6 mg/ml | Sigma | E2758 | 5 ul |
| Progesteron 0.6 mg/ml | Sigma | P8783 | 10 ul |
| Stem Pro Supplement | Invitrogen | 10639-011 | 2,6 ml |
| EGF, 200 µg/ml | Sigma | E9644 | 10 µL |
| bFGF, 10 µg/ml | Sigma | F0291 | 100 µL |
| GDNF, 10 µg/ml | Sigma | G1777 | 100 µL |
| LIF, 10 µg/ml | Chemicon | LIF1010 | 100 µL |
| FCS | Invitrogen | 10106-169 | 250 µL |
| Pen/strep | Invitrogen | 15140122 | 500 µL |

Supplemental table 2 Taqman primer assays for qPCR

| Genes | Company | Product Nr. |
| --- | --- | --- |
| LIN28A | Thermo Fisher Scientific | Hs01552403_g1 |
| GAGE1 | Thermo Fisher Scientific | Hs01695930_s1 |
| GAPDH | Thermo Fisher Scientific | Hs025978991_g1 |
| PLZF | Thermo Fisher Scientific | Hs00957433_m1 |
| ITGB1 | Thermo Fisher Scientific | Hs00559595_m1 |
| ITGA6 | Thermo Fisher Scientific | Hs01041011_m1 |
| FSP1 | Thermo Fisher Scientific | Hs00210845_m1 |
| AMH | Thermo Fisher Scientific | Hs00174915_m1 |
| INLS3 | Thermo Fisher Scientific | Hs01895076_s1 |
